# A Convenient Synthesis of Type A Procyanidins

**DOI:** 10.3390/molecules14020807

**Published:** 2009-02-17

**Authors:** George A. Kraus, Yi Yuan, Aaron Kempema

**Affiliations:** Department of Chemistry, Iowa State University, Ames, IA 50011, USA

**Keywords:** Procyanidins, Catechin, Synthesis.

## Abstract

Type A procyanidins can be synthesized in good yields from the condensation of benzopyrilium salts **8** and either catechin or phloroglucinol.

## Introduction

The prevention and control of Type 2 diabetes is a major human healthcare objective. The pathogenesis of this disease has been studied by several researchers [1]. Diet has been shown to play a definite role in the onset of Type 2 diabetes, and the foods typically consumed in the United States appear to increase the incidence of diabetes [2]. A Finnish study showed that Type 2 diabetes could be prevented by changes in the lifestyles of high-risk subjects [3]. Another recent study concluded that Type 2 diabetes could be prevented or delayed through lifestyle modifications or through pharmacologic interventions [4]. The relatively high sugar and fat content of American diets is likely to increase the incidence of diabetes, but the low intake of traditional herbs, spices, and other plant products may also be a factor. 

Plants may not only be important for the control of Type 2 diabetes but also for its prevention, especially for people with elevated levels of blood glucose and glucose intolerance who have a greater risk of developing diabetes. Common spices such as cinnamon, cloves, and bay leaves display insulin-potentiating activity *in vitro* [5]. It was hypothesized that these spices might also have high chromium concentrations, because biologically active forms of chromium potentiate insulin activity. However, there were no correlations between total chromium concentrations and insulin potentiating activity in these plant products. 

From an aqueous extract of commercial cinnamon, Anderson and coworkers have identified polyphenolic polymers that increase glucose metabolism roughly 20-fold *in vitro* in the epididymal fat cell assay [6]. Additionally, approximately 50 plant extracts have also been investigated in this assay, and none have shown activity equal to that of cinnamon [7].

Recently, Anderson and coworkers extracted cinnamon samples with acetic acid and ethanol and isolated fractions with insulin-enhancing activity using HPLC [8,9,10]. Spectral analysis indicated the presence of type A procyanidins. The procyanidins encompass dimers, trimers and higher oligomers composed of catechin (**1**) and epicatechin (**2**). In procyanidins, the B series of compounds is characterized by one interflavanoid linkage (one C-C bond) and is exemplified by procyanidins B2 (**3**) and B3 (**4**). The A series of compounds contains two interflavanoid linkages (one C-C bond and one C-O bond). Procyanidins A1 (**5**) and A2 (**6**) are active compounds from this series. The C series consists of trimers of catechin or epicatechin and is represented by compound **7**. 

**Figure 1:**
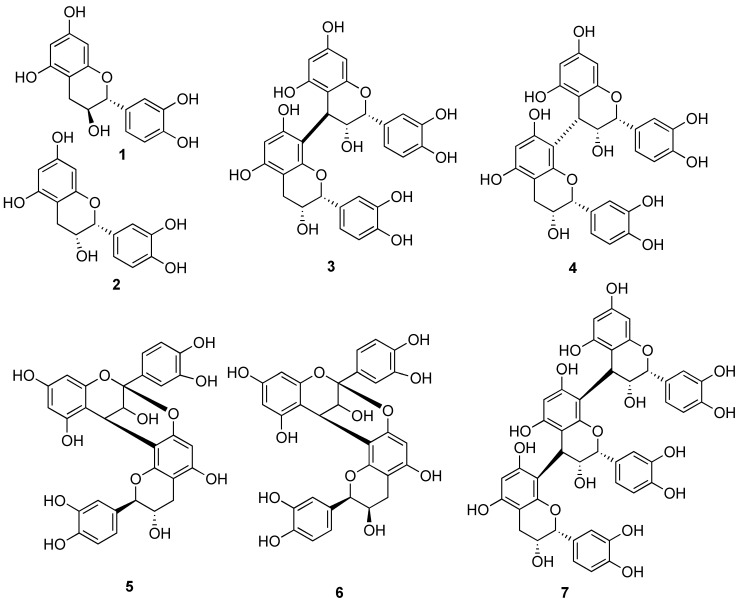
Structures of Procyanidins.

The fractions containing these A-type procyanidins were shown to have insulin-enhancing biological activity in an *in vitro* assay measuring the insulin-dependent effects on glucose metabolism [6]. These same compounds have been shown to inhibit phosphotyrosine phosphatases in the insulin-receptor domain and to activate insulin receptor kinase, and to function as a mimetic for insulin in 3T3-L1 adipocytes [11]. Water-soluble polymeric polyphenols from cinnamon have also been shown to inhibit cell proliferation and to alter cell cycle distribution patterns of hematologic tumor cell lines [12].

## Results and Discussion

**Scheme 1:**
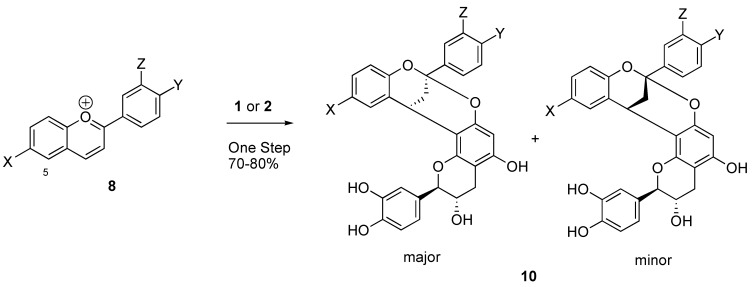
Synthetic route.

Type A procyanidins are difficult to synthesize. Morimoto prepared procyanidins A in low yield by the oxidation of procyanidin B2 with hydrogen peroxide in the presence of sodium bicarbonate [13]. Pomilo reacted a flavylium perchloride with (+)-catechin in aqueous methanol to yield a compound in only 2-3% yield [14]. Therefore, there is a continuing need for methods to prepare polyphenols such as those derived from cinnamon. Recently Pettus prepared a type A procyanidin (diinsininone) using microwave chemistry at 120 °C [15]. Our route to type A procyanidins is illustrated below in Scheme 1. Although it utilizes starting materials similar to those used by Pomilo and by Morimoto, our optimized conditions provide dramatically improved yields [16]. 

The synthetic route shown in Scheme 1 permits the preparation of a variety of procyanidin structures. In this paper we will describe our synthesis of compounds of general structure **10**, with X, Y and Z as either electron-donating or electron-withdrawing groups. The benzopyrilium salts **8** were generated in yields ranging from 70-90% from the corresponding salicylaldehyde and acetophenone by a standard acid catalyzed cyclization. For example, the compound **8** wherein X and Y are hydroxyl groups and Z is a hydrogen was prepared in 85% yield by condensing salicylaldehyde and 3,4-dihydroxyacetophenone [17]. The reaction of the benzopyrilium salts with **1** to produce **10** was conducted under anhydrous conditions in methanol at temperatures ranging from ambient temperature to 50 °C. The results of our research are compiled below in Table 1. The products were mixtures of two diastereomers in ratios of 1.2:1 to 1.5:1. Molecular models show that the methine proton of the benzylic ether (C-2 of catechin) in the minor isomer should be deshielded relative to the corresponding methine of the major isomer. However, the actual difference in chemical shifts is small and thus the structure assignments are tentative. This is observed for **10A - 10F** and is the basis for assignment of the structures of the diastereomers. As indicated by Table 1, a variety of substituent patterns are possible. The only limitation we found was that substitution at position 5 in compound **8** dramatically reduced the rate of cyclization. The rationale for the improved yields is unclear and may reflect the importance of limiting the amount of water present during the reaction. 

**Table 1:** Reactions of **1** with Benzopyrilium Salts. 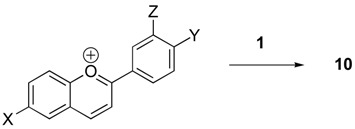

X	Y	Z	Yield (%)	dr	10
H	H	H	82	1.5	**10A**
H	OH	H	79	1.2	**10B**
H	OH	OH	89	1.5	**10C**
NO_2_	OH	H	83	1.5	**10D**
NO_2_	OH	OH	76	1.5	**10E**
H	OCH_3_	OCH_3_	83	1.2	**10F**

The salts also reacted effectively with phloroglucinol (**9**). In this case only a single diastereomer could be formed. Compounds **11** and **12** were produced in 76% and 80% yields, respectively. 

**Scheme 2:**
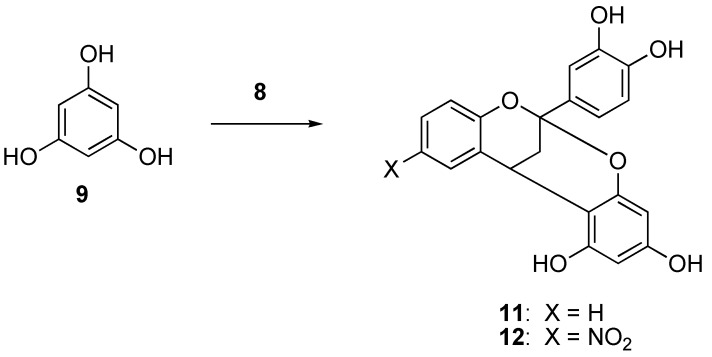
Synthesis of phloroglucinol adducts.

In summary, type A procyanidins **10** - **12** are readily synthesized from benzopyrilium salts **8** and either catechin or phloroglucinol. The experimental conditions are operationally convenient and the reaction can be scaled up to produce gram quantities of type A procyanidins. 

## Experimental

### General

Unless stated otherwise, all reactions were magnetically stirred and monitored by thin-layer chromatography (TLC) using 0.25 mm precoated silica gel F254 plates (Sigma-Aldrich). Column or flash chromatography was performed with the indicated solvents using silica gel (230-400 mesh) purchased from Dynamic Adsorbents, LLC. All melting points were obtained on a Laboratory Devices capillary melting point apparatus and are uncorrected. ^1^H- and ^13^C-NMR spectra were recorded on a Bruker VXR-400 (400 MHz) spectrometer. Chemical shifts are reported relative to internal chloroform (^1^H, 7.26 ppm; ^13^C, 77.23 ppm). High resolution mass spectra were performed at the Iowa State University Mass Spectrometry Laboratory.

### General synthesis of type A procyanidins **10A-10F**, **11** and **12**

To a solution of (+)-catechin hydrate (925 mg, 3.0 mmol) or phloroglucinol (378 mg, 3.0 mmol) in methanol (65 mL) was added flavylium salt (920 mg, 3.0 mmol) at room temperature (rt). The solution was stirred at rt for 15 h, concentrated and adsorbed on approximately a gram of silica gel. The crude product was purified by column chromatography on silica gel using hexanes-ethyl acetate-methanol (2:1:0 – 1:1.5:0.005) as eluent. Gram quantities of product were collected in yields ranging from 70-89%. The products **10A-10F** contain two stereoisomers in a ratio indicated by the proton NMR spectrum. Polar diastereomers were separated using silica gel preparative TLC using 10:1 ethyl acetate: hexanes. 

*(2R,3S,8S,14S)-2-(3,4-Dihydroxyphenyl)-8-phenyl-3,4-dihydro-2H,14H-8,14-methanochromeno[7,8-d][1,3]benzodioxocin-3,5-diol and (2R,3S,8R,14R)-2-(3,4-dihydroxyphenyl)-8-phenyl-3,4-dihydro-2H,14H-8,14-methanochromeno[7,8-d][1,3]benzodioxocin-3,5-diol* (**10A**). Yield 82%. Major isomer (60%). ^1^H-NMR (400 MHz, CD_3_OD) 7.70-7.61 (m, 2H), 7.42-7.31 (m, 3H), 7.14 (dd, *J* = 7.6, 1.6 Hz, 1H), 7.10- 6.98 (m, 2H), 6.93-6.72 (m, 4H), 6.09 (s, 1H), 4.58 (d, *J* = 8.0 Hz, 1H), 4.33 (t, *J* = 3.0 Hz, 1H), 4.02 (m, 1H), 2.95 (dd, *J* = 16.4, 5.6 Hz, 1H), 2.51 (dd, *J* = 16.4, 8.0 Hz, 1H), 2.22 (dd, *J* = 13.2, 3.0 Hz, 1H), 2.18 (dd, *J* = 13.2, 3.0 Hz, 1H); ^13^C-NMR (100 MHz, CD_3_OD)  155.7, 153.7, 152.7, 152.0, 146.4, 146.3, 146.3, 143.3, 132.1, 129.6, 129.2, 129.1, 128.9, 128.6, 128.3, 126.7, 122.0, 120.2, 116.8, 116.3, 115.5, 106.9, 102.8, 99.9, 96.3, 823.0, 68.7, 34.5, 29.3, 28.0; HRMS (EI) m/z 496.1530 (M, 496.1522 calcd for C_30_H_24_O_7_); MS(EI) m/z 496 (11), 207 (100), 178 (39), 152 (58), 131 (70). Minor isomer (40%). ^1^H-NMR (400 MHz, CD_3_OD)   7.70-7.61 (m, 2H), 7.42-7.31 (m, 3H), 7.26 (dd, *J* = 7.6, 1.6 Hz, 1H), 7.10- 6.98 (m, 2H), 6.93-6.72 (m, 4H), 6.10 (s, 1H), 4.71 (d, *J* = 8.0 Hz, 1H), 4.27 (t, *J* = 3.0 Hz, 1H), 3.96 (m, 1H), 2.87 (dd, *J* = 16.4, 5.6 Hz, 1H), 2.55 (dd, *J* = 16.4, 8.0 Hz, 1H), 2.16 (dd, *J* = 13.2, 3.0 Hz, 1H), 2.10 (dd, *J* = 13.2, 3.0 Hz, 1H); ^13^C-NMR (100 MHz, CD_3_OD)   155.8, 153.6, 152.5, 152.0, 146.4, 146.3, 146.3, 143.3, 132.2, 129.6, 129.2, 129.1, 128.9, 128.6, 128.4, 126.7, 122.0, 120.4, 116.8, 116.0, 115.5, 106.8, 102.5, 99.9, 96.2, 83.5, 68.8, 34.6, 28.6, 27.9.

*(2R,3S,8S,14S)-2-(3,4-Dihydroxyphenyl)-8-(4-hydroxyphenyl)-3,4-dihydro-2H,14H-8,14-methano-chromeno[7,8-d][1,3]benzodioxocin-3,5-diol and (2R,3S,8R,14R)-2-(3,4-dihydroxyphenyl)-8-(4-hydroxyphenyl)-3,4-dihydro-2H,14H-8,14 methanochromeno [7,8d][1,3] benzodioxocin-3,5-diol* (**10B**) Yield 78%. Major isomer (55%): ^1^H-NMR (400 MHz, CD_3_OD)   7.54-7.48 (m, 2H), 7.12 (d, *J* = 7.2 Hz, 1H), 7.09 - 6.70 (m, 8H), 6.11 (s, 1H), 4.59 (d, *J* = 8.4 Hz, 1H), 4.35 (t, *J* = 3.0 Hz, 1H), 3.98 (m, 1H), 3.01 (dd, *J* = 16.4, 5.6 Hz, 1H), 2.52 (dd, *J* = 16.4, 8.4 Hz, 1H), 2.25 (dd, *J* = 13.2, 3.0 Hz, 1H), 2.16 (dd, *J* = 13.2, 3.0 Hz, 1H). Minor isomer (45%): ^1^H NMR (400 MHz, CD_3_OD)   7.54-7.48 (m, 2H), 7.27(d, *J* = 7.2 Hz, 1H), 7.09-6.70 (m, 8H), 6.12 (s, 1H), 4.71 (d, *J* = 8.4 Hz, 1H), 4.31 (t, *J* = 3.0 Hz, 1H), 4.04 (m, 1H), 2.92 (dd, *J* = 16.4, 5.6 Hz, 1H), 2.57 (dd, *J* = 16.4, 8.4 Hz, 1H), 2.19 (dd, *J* = 13.2, 3.0 Hz, 1H), 2.13 (dd, *J* = 13.2, 3.0 Hz, 1H).

*(2R,3S,8S,14S)-2-(3,4-Dihydroxyphenyl)-8-(3,4-dihydroxyphenyl)-3,4-dihydro-2H,14H-8,14-methanochromeno[7,8-d][1,3]benzodioxocin-3,5-diol and (2R,3S,8R,14R)-2-(3,4-dihydroxyphenyl)-8-(3,4-dihydroxyphenyl)-3,4-dihydro-2H,14H-8,14-methanochromeno[7,8-d][1,3]-benzodioxocin-3,5-diol* (**10C**). Yield 89%. Major isomer (60%): ^1^H-NMR (400 MHz, CD_3_CN)   7.50-6.75 (m, 10H), 6.09 (s, 1H), 4.64 (d, *J* = 8.0 Hz, 1H), 4.33 (br, 1H), 4.06 – 3.92 (m, 1H), 2.93 – 2.72 (m, 1H), 2.57 – 2.42 (m, 1H), 2.32 – 2.08 (m, 2H). Minor isomer (40%): ^1^H-NMR (400 MHz, CD_3_CN)   7.50 -6.75 (m, 10H), 6.10 (s, 1H), 4.73 (d, *J* = 8.0 Hz, 1H), 4.25 (br, 1H), 4.06 – 3.92 (m, 1H), 2.93 – 2.72 (m, 1H), 2.57 – 2.42 (m, 1H), 2.32 – 2.08 (m, 2H).

*(2R,3S,8S,14S)-2-(3,4-Dihydroxyphenyl)-8-(4-hydroxyphenyl)-12-nitro-3,4-dihydro-2H,14H-8,14-methanochromeno[7,8-d][1,3]benzodioxocin-3,5-diol and (2R,3S,8R,14R)-2-(3,4-dihydroxyphenyl)-8-(4-hydroxyphenyl)-12-nitro-3,4-dihydro-2H,14H-8,14-methanochromeno[7,8-d][1,3]benzodioxocin-3,5-diol* (**10D**). Yield 83%. Major isomer (60%): ^1^H-NMR (400 MHz, CD_3_OD)   7.99 (d, *J* = 2.8 Hz, 1H), 7.77 (dd, *J* = 8.8, 2.8 Hz, 1H), 7.52-7.40 (m, 2H), 7.08-6.78 (m, 6H), 6.07 (s, 1H), 4.59 (d, *J* = 8.0 Hz, 1H), 4.34 (t, *J* = 2.8 Hz, 1H), 4.11 (ddd, *J* = 16.4, 8.0, 5.6 Hz, 1H), 2.95 (dd, *J* = 16.4, 5.6 Hz, 1H), 2.49 (dd, *J* = 16.4, 8.4 Hz, 1H), 2.26-2.01(m, 2H); ^13^C-NMR (100 MHz, CD_3_OD)   159.3, 158.9, 152.7, 151.5, 146.4, 146.2, 142.4, 133.1, 131.5, 130.0, 127.1, 124.6, 124.4, 124.2, 124.1, 121.1, 117.3, 119.6, 116.4, 115.9, 115.5, 105.6, 103.1, 100.9, 96.3, 83.0, 68.1, 33.3, 29.1, 27.9. Minor isomer (40%): ^1^H-NMR (400 MHz, CD_3_OD)   8.14 (d, *J* = 2.8 Hz, 1H), 7.84 (dd, *J* = 8.8, 2.8 Hz, 1H), 7.52-7.40 (m, 2H), 7.08-6.78 (m, 6H), 6.07 (s, 1H), 4.68 (d, *J* = 8.0 Hz, 1H), 4.27 (t, *J* = 2.8 Hz, 1H), 3.94 (ddd, *J* = 16.4, 8.0, 5.6 Hz, 1H), 2.88 (dd, *J* = 16.4, 5.6 Hz, 1H), 2.54 (dd, *J* = 16.4, 8.4 Hz, 1H), 2.26-2.01(m, 2H); ^13^C-NMR (100 MHz, CD_3_OD)   159.4, 158.9, 156.1, 152.5, 151.5, 146.2, 145.9, 142.4, 133.1, 131.0, 129.8, 127.1, 124.6, 124.4, 124.1, 119.6, 121.1, 117.4, 116.8, 116.3, 115.9, 105.4, 103.0, 100.9, 96.4, 83.6, 68.8, 33.4, 28.8, 27.9.

*(2R,3S,8S,14S)-2,8-bis-(3,4-Dihydroxyphenyl)-12-nitro-3,4-dihydro-2H,14H-8,14-methanochromeno-[7,8-d][1,3]benzodioxocin-3,5-diol and (2R,3S,8R,14R)-2,8-bis-(3,4-dihydroxyphenyl)-12-nitro-3,4-dihydro-2H,14H-8,14-methanochromeno[7,8-d][1,3]benzodioxocin-3,5-diol* (**10E**). Yield 76%. Major isomer (60%): ^1^H-NMR (400 MHz, CD_3_OD)   8.01 (br, 1H), 7.94 (dd, *J* = 8.8, 2.0 Hz, 1H), 7.18-6.78 (m, 7H), 6.09 (s, 1H), 4.61 (d, *J* = 7.6 Hz, 1H), 4.37 (br, 1H), 4.14 (m, 1H), 2.95 (dd, *J* = 16.4, 5.2 Hz, 1H), 2.51 (dd, *J* = 16.4, 8.4 Hz, 1H), 2.32-2.06(m, 2H); ^13^C-NMR (100 MHz, CD_3_OD)   159.4, 156.2, 152.7, 151.5, 146.8, 146.2, 145.9, 142.5, 133.8, 131.5, 130.1, 124.4, 124.3, 121.0, 118.2, 117.3, 116.8, 116.4, 116.2, 115.9, 115.4, 114.1, 105.6, 103.1, 100.8, 83.5, 68.1, 33.5, 29.1, 27.9. Minor isomer (40%): ^1^H-NMR (400 MHz, CD_3_OD)   8.16 (br, 1H), 7.91 (dd, *J* = 8.8, 2.0 Hz, 1H), 7.18-6.78 (m, 7H), 6.09 (s, 1H), 4.70 (d, *J* = 7.6 Hz, 1H), 4.32 (br, 1H), 3.95 (m, 1H), 2.89(dd, *J* = 16.4, 5.2 Hz, 1H), 2.55 (dd, *J* = 16.4, 8.4 Hz, 1H), 2.32-2.06(m, 2H); ^13^C-NMR (100 MHz, CD_3_OD)   159.5, 156.2, 152.5, 151.5, 146.4, 146.2, 145.9, 142.4, 133.8, 131.9, 129.9, 124.6, 124.1, 119.5, 118.3, 117.4, 116.4, 116.2, 115.9, 115.4, 114.1, 105.4, 102.9, 100.8, 96.3, 83.6, 68.8, 33.6, 28.7, 27.9.

*(2R,3S,8R,14R)-2-(3,4-Dihydroxyphenyl)-8-(3,4-dimethoxyphenyl)-3,4-dihydro-2H,14H-8,14-methanochromeno[7,8-d][1,3]benzodioxocin-3,5-diol and (2R,3S,8S,14S)-2-(3,4-dihydroxyphenyl)-8-(3,4-dimethoxyphenyl)-3,4-dihydro-2H,14H-8,14-methanochromeno[7,8-d][1,3]benzodioxocin-3,5-diol:* (**10F**). Yield 83%. Major isomer (55%): ^1^H-NMR (400 MHz, CD_3_COCD_3_)   7.38-6.74 (m, 10H), 6.16 (s, 1H), 4.62 (d, *J* = 8.0 Hz, 1H), 4.38 (t, *J* = 3.0 Hz, 1H), 4.13 (m, 1H), 3.85(m, 3H), 3.83(m, 3H), 3.03 (dd, *J* = 16.4, 5.6 Hz, 1H), 2.57 (dd, J = 16.4, 8.0 Hz, 1H), 2.34 (dd, *J* = 13.6, 3.0 Hz, 1H), 2.25 (dd, *J* = 13.6, 3.0 Hz, 1H); ^13^C-NMR (100 MHz, CD_3_COCD_3_)   156.2, 154.3, 153.4, 152.7, 150.9, 146.8, 146.6, 146.5, 133.1, 132.8, 129.2, 129.0, 122.6, 121.2, 120.0, 116.9, 116.3, 116.1, 113.1, 107.6, 103.6, 100.4, 97.1, 83.7, 69.3, 57.2, 57.1, 34.9, 29.9, 28.5. Minor isomer (45%): ^1^H-NMR (400 MHz, CD_3_COCD_3_)   7.38-6.74 (m, 10H), 6.18 (s, 1H), 4.73 (d, *J* = 8.0 Hz, 1H), 4.30 (t, *J* = 3.0 Hz, 1H), 4.01 (m, 1H), 3.84(m, 3H), 3.83(m, 3H), 2.97 (dd, *J* = 16.4, 5.2 Hz, 1H), 2.61 (dd, *J* = 16.4, 8.0 Hz, 1H), 2.28 (dd, *J* = 13.6, 3.0 Hz, 1H), 2.22 (dd, *J* = 13.6, 3.0 Hz, 1H); ^13^C-NMR (100 MHz, CD_3_COCD_3_)   156.2, 154.3, 153.2, 152.7, 151.5, 146.8, 146.7, 140.5, 133.0, 129.7, 129.5, 129.0, 121.3, 117.6, 116.7, 116.7, 116.4, 111.8, 107.5, 103.4, 100.3, 97.1, 96.4, 84.4, 69.1, 57.2, 57.1, 35.0, 29.7, 28.5.

*(±)-6-(3,4-Dihydroxyphenyl)-12H-6,12-methanodibenzo[d,g][1,3]dioxocin-1,3-diol* (**11**). Yield 76%. ^1^H-NMR (400 MHz, CD_3_COCD_3_)   7.42 (dd, *J* = 7.2, 1.2 Hz, 1H), 7.31 (m, 2H), 7.10 (ddd, *J* = 10, 8.4, 2.0 Hz, 1H), 7.01 (d, 8.4 Hz, 1H), 6.92 (d, *J* = 8.4 Hz, 1H), 6.87 (ddd, *J* = 8.4, 7.2, 0.8 Hz, 1H), 6.05 (d, *J* = 2.4 Hz, 1H), 6.02(d, *J* = 2.4 Hz, 1H), 4.42 (t, *J* = 2.8 Hz, 1H), 2.31 (t, *J* = 2.8 Hz, 2H).

*(±)-6-(3,4-Dihydroxyphenyl)-10-nitro-12H-6,12-methanodibenzo[d,g][1,3]dioxocin-1,3-diol* (**12**). Yield 80%: ^1^H-NMR (400 MHz, CD_3_COCD_3_)   8.30 (d, *J* = 2.8 Hz, 1H), 8.02 (dd, *J* = 8.8, 2.8 Hz, 1H), 7.25 (d, *J* = 2.8 Hz, 1H), 7.10 (m, 2H), 6.92 (d, *J* = 8.8 Hz, 1H), 6.10 (d, *J* = 2.8 Hz, 1H), 6.05 (d, *J* = 2.8 Hz, 1H), 4.53 (t, *J* = 2.8 Hz, 1H), 2.39 (t, *J* = 2.8 Hz, 2H); ^13^C-NMR (100 MHz, CD_3_COCD_3_)   159.9, 159.2, 156.6, 154.6, 147.4, 146.4, 143.1, 134.5, 130.8, 125.1, 124.9, 119.1, 118.4, 116.7, 114.9, 106.3, 101.4, 96.9, 96.3, 34.2, 28.4.
